# Regulating Emotions during Difficult Multiattribute Decision Making: The Role of Pre-Decisional Coherence Shifting

**DOI:** 10.1371/journal.pone.0150873

**Published:** 2016-03-17

**Authors:** Stephanie M. Carpenter, J. Frank Yates, Stephanie D. Preston, Lydia Chen

**Affiliations:** 1 Department of Psychology, University of Michigan, Ann Arbor, Michigan, United States of America; 2 Department of Marketing, Ross School of Business, University of Michigan, Ann Arbor, Michigan, United States of America; Technion Israel Institute of Technology, ISRAEL

## Abstract

Almost all real-life decisions entail attribute conflict; every serious choice alternative is better than its competitors on some attribute dimensions but worse on others. In pre-decisional “coherence shifting,” the decision maker gradually softens that conflict psychologically to the point where one alternative is seen as dominant over its competitors, or nearly so. Specifically, weaknesses of the eventually chosen alternative come to be perceived as less severe and less important while its strengths seem more desirable and significant. The research described here demonstrates that difficult multiattribute decision problems are aversive and that pre-decisional coherence shifting aids individuals in regulating that emotional discomfort. Across three studies, attribute conflict was confirmed to be aversive (Study 1), and skin conductance responses and ratings of decision difficulty both decreased in participants who coherence shifted (Study 2). Coherence shifting was also diminished among decision makers who were depleted of regulatory resources, known to be required for common emotion regulation mechanisms. Further, coherence shifting was shown to be relatively common among people who reported strong suppression tendencies in everyday emotion regulation (Study 3). Overall, the data suggest that, at least in part, coherence shifting serves as a tool that helps decision makers manage the pre-decisional discomfort generated by attribute conflict. Theoretical and practical implications are discussed.

## Introduction

Picture yourself as a college senior facing the following decision problem (cf. [1]): You must choose between two job offers that differ from each other on four attribute dimensions. As described in Table 1, Job Offer 1 is for an entry-level marketing position at the “Splendor” department store chain, and Job Offer 2 is for a similar opportunity at the “Bonnie’s Best” (BB) chain. You recognize that the Splendor offer is superior to the BB offer with respect to the office (private vs. cubicle) and commute time (18 vs. 40 min) dimensions. On the other hand, the BB offer is better on salary ($800 above the industry average of $40,000 vs. $600 less than that average) and vacation package (superior vs. minimal time off). You have already concluded that the offers are comparable with respect to every other attribute dimension that you care about, and therefore you ignore those other factors.

**Table 1:** Decision Matrix for Hypothetical Job Offers.

Attribute Dimension	Job Offer	
	1—Splendor	2—Bonnie’s Best (B)
	*Attribute*	*Attribute*
Office	Private	Cubicle
Commute	18 minutes	40 minutes
Salary	$39,400 (below $40,000 industry standard)	$40,800 (above $40,000 industry standard)
Vacation	Minimal time off	Superior package

Note. Adapted from Simon et al. [1].

Your Splendor/Bonnie’s Best conundrum illustrates a fundamental concern in decision scholarship: “attribute conflict,” that is, the absence of dominance (a situation in which one alternative is at least as good as every competitor with respect to every attribute dimension and better on at least one of those dimensions). This challenge was also highlighted in Benjamin Franklin’s famous 1772 letter to his friend Joseph Priestly concerning “moral or prudential algebra,” in which he recommended a pro-vs.-con list approach to tough decision problems [2]. In nearly every real-life decision situation, deliberations eventually reach a point where attribute conflict becomes apparent. As in the Splendor/BB scenario, if one attribute dimension (e.g., office) favors Alternative A, then one or more other dimensions (e.g., salary) favor some competing alternative. A key challenge in descriptive decision scholarship has been to determine *how* people resolve attribute conflict in actual practice, thereby arriving at their ultimate choices. Another, less common, aim has been to understand *why* decision makers resolve such conflict as they do. The latter is our focus here. In particular, we report three studies whose results are consistent with the following conclusions: (a) attribute conflict generates aversive emotions (including stress) and (b) that decision makers invoke a means of addressing attribute conflict that simultaneously relieves the decision maker’s discomfort as well as points toward a highly satisfying decision alternative.

### Coherence Shifting: A Special Way That People Resolve Attribute Conflict

Many traditional decision theoretic approaches to resolving attribute conflict (implicitly, even Ben Franklin’s approach) rest on “weighted additive” (WADD) value representations such as the following for the Splendor/BB problem (cf. [3]):

$$T(Splendor)=W_{Off}\times A_{Off}(Splendor)+W_{Comm}\times A_{Comm}(Splendor)+W_{Sal}\times A_{Sal}(Splendor)+W_{Vac}\times A_{Vac}(Splendor)$$ (1)

T(Splendor), Eq 1, can be interpreted as a score representing the overall, total appeal of the Splendor job offer in the situation described above. W_Off_ is your importance weight for the office dimension, and A_Off_(Splendor) is a measure of your appraisal of the Splendor job offer on that feature dimension—essentially how much you like Splendor’s private office. The corresponding terms with the Comm, Sal, and Vac subscripts have similar interpretations for the commute, salary, and vacation dimensions, respectively. Note that a parallel score, T(Bonnie’s Best), could be derived for the competing job offer at BB. The final element of the traditional scheme for resolving attribute conflict is the “decision rule,” which specifies that the decision maker select the alternative that has the best overall score. Thus, you would choose Splendor if T(Splendor) > T(Bonnie’s Best) or instead pick Bonnie’s Best if the opposite were true (and you would be indifferent if T(Splendor) = T(Bonnie’s Best)). It is important to recognize that the magnitude of the difference between T(Splendor) and T(Bonnie’s Best) does not matter, only the direction. This is related to the fact that the *strength* of attribute conflict plays no role in the decision process. Leaving aside considerations of assessment error, if T(Spendor) > T(Bonnie’s Best), the decision maker chooses Spendor.

Now return to your job choice deliberations. Suppose that something—perhaps your encountering the Splendor option first—induces you to lean slightly toward that offer (cf. [4]). Table 2 depicts two snapshots of your early (Time 1) and later (Time 2) deliberations in the decision episode. These snapshots include numerical representations of your attribute appraisals and dimension weights, using the kinds of ratings elicited by Simon et al. [1] as well as the WADD value function. Notice that the attribute weights and appraisals have yielded overall scores such that, at Time 1, T(Splendor)_Time 1_ = +2 and T(Bonnie’s Best)_Time 1_ = -2, slightly in favor of Splendor. Later, at Time 2, things have changed markedly. Observe that T(Splendor)_Time 2_ = +18 and T(Bonnie’s Best)_Time 2_ = -23. Also observe *how* this occurred. First, your weights for the dimensions (e.g., commute) on which your initially favored alternative (Splendor) was strong have increased, while those for dimensions on which it was weak (e.g., salary) have decreased. In addition, you now appraise more favorably than before those Splendor attributes that are objectively stronger than their BB counterparts (e.g., small office versus noisy cubicle). You also do the opposite for BB attributes that are stronger than the corresponding Splendor attributes (e.g., salary, vacation), seeing them as less favorable than you did initially. That is, you have shifted your appraisals and importance weights to be even more “coherent” with your initial choice leaning than they were at the outset. Put another way, the severity of the prior attribute conflict has been softened; psychologically, at least, you are now closer to experiencing a dominating alternative (cf. [5]). Note that this process differs significantly from the traditional view in that it highlights the notion that the simple marginal superiority of one alternative over another is not enough: the goal is simply to establish a clear best option, perhaps even a dominant one.

**Table 2:** Hypothetical Time 1 → Time 2 Coherence Shifts for Decision Maker with Initial Leaning Toward Job Offer 1—Splendor.

Attribute Dimension		Job Offer			
		1—Splendor		2—Bonnie’s Best (B)	
	*Weight*^a^	*Attribute*	*Appraisal*^b^	*Attribute*	*Appraisal*
Office	*4 → 5*	Private	*+3 → +4*	Cubicle	*-2 → -3*
Commute	*2 → 3*	18 minutes	*-1 → 0*	40 minutes	*-2 → -4*
Salary	*3 → 2*	$39,400 (below $40,000 industry standard)	*-2 → -1*	$40,800 (above $40,000 industry standard)	*+2 → +*
Vacation	*2 → 1*	Minimal time off	*-1 → 0*	Superior package	*+2 → +1*
Overall Score		*+2 → +18*		*-2 → -23*	

Note. Adapted from Simon et al. [1].

^a^Attribute dimension importance weight scale: 0 (no weight) … 8 (maximum weight)

^b^Attribute appraisal rating scale: -5 (highly undesirable) … +5 (highly desirable)

In relatively recent times, several investigators [1, 6, 7] have demonstrated that this “coherence shifting” is a reliable phenomenon, one with exceptional (and generally unacknowledged) importance for decision scholarship. It amounts to a means of resolving attribute conflict that is fundamentally different from the approach posited in traditional decision theories. It does not assume that the decision maker’s attribute assessments and dimensional importance weights are either fixed or stochastic. Rather, they shift systematically during the course of a decision episode. The most significant unmet challenge is to explain why this happens.

Investigators have proposed numerous drivers of coherence shifting. In one form or another, the most commonly discussed proposals suggest that coherence shifting at least partly reflects the decision maker’s aim of reducing the cognitive effort expended during the decision process [5, 8, 9]. One version of this idea, suggested by Simon et al. [1] as well as Glöckner, Betsch, and Schindler [10], entails a “constraint satisfaction” mechanism. This mechanism is presumed to be an automatic, Gestalt perception-like process that rapidly makes sense of the decision maker’s situation [10]. This allows for quick, efficient, and confident action since most, if not all, of the pertinent considerations are consistent with the selection of one particular alternative. Russo, Carlson, Meloy, and Yong [11] proposed an especially interesting extension of this idea. Specifically, they suggested that consistency, in and of itself, serves as a goal for decision makers, and pre-decisional coherence shifting helps decision makers achieve that goal. The results described by Russo et al. agree with such an interpretation, and also exclude the goals of effort and efficiency as drivers of pre-decisional shifting, leaving other possibilities to be examined. Finally, Meloy [12] focused on positive mood and suggested that the maintenance of a positive mood state might be another motivation for pre-decision distortion behavior.

Henry Montgomery and Ola Svenson have offered closely related explanations for coherence shifting. Montgomery’s ([13], p. 343) “Search for Dominance Structure Model” maintains that decision making is a search for good arguments favoring one course of action over its competitors. Further, the most powerful argument one can offer is that the focal option dominates those competitors. Thus, once the need for making a decision is recognized, the decision process reduces to a search for dominance. The model simply assumes that the decision maker “will attempt to create dominance by changing his or her representation of the decision situation such that one alternative becomes dominant” ([13] p. 343); that assumption is not explicitly rationalized. In effect, this is a proposal that coherence shifting is driven by the sheer need to decide convincingly.

In his “Differentiation and Consolidation Theory,” Svenson [14–15] goes further. “Differentiation” refers to the procedures (including searching for, creating, or reinterpreting alternatives to the point where they are seen as dominant). Diff Con Theory asserts that these operations do not cease when dominance, and hence a choice, is achieved. They continue during the “consolidation” of support for that decision. The result is that the dominance of the chosen alternative becomes stronger and stronger. This means that the decision maker is unlikely to be tempted to reopen the decision episode because an attractive alternative comes along. Underneath this Diff Con proposal are “safety” and “stability” motives ([15], p. 318). That is, the decision maker should feel confident that he or she has chosen a “safe,” dominant alternative. Moreover, that alternative is seen to be so strong that the decision maker is unlikely to be constantly revisiting that choice.

### Emotion Regulation: A Proposed Reason That People Coherence Shift

All of the various proposed contributors to coherence shifting are plausible and in some instances are supported by sound evidence. However, the present research was predicated on doubt that such proposals constitute the whole story. Based partly on interviews and think-aloud protocols of people making multiattribute choices, we considered a distinct, additional role for emotions. We conjectured that attribute conflict generates emotional discomfort, which in turn motivates efforts to reduce that discomfort. Undertaking coherence shifting would do two things for the decision maker. First, because it softens attribute conflict, it would diminish discomfort. Second, since coherence shifting tends to make one option appear (almost) uniformly attractive, it essentially solves the decision problem, too. That is, even if not consciously, some individuals are expected to use coherence shifting as a tool for minimizing unpleasant affect. Others, who coherence shift minimally, if at all, are not relieved of that discomfort.

Prior research has suggested that tradeoff dilemmas, implicit in attribute conflict, should be expected to produce negative affect, particularly if a person perceives that he or she does not have the resources to cope with those tradeoffs [16]. As proposed by Janis and Mann ([17] p. 46), such feelings are fed by the fact such tradeoffs imply the likelihood of losses, absolute or relative. We argue that difficult decisions involving attribute conflict are especially likely to be emotional, and that coherence shifting is a strategy that effectively aids people in managing these negative emotions. At first blush, coherence shifting may also seem to be the same as the post-decisional spreading of alternatives that is often attributed to cognitive dissonance reduction [18–19]. As defined by Festinger [19–20], cognitive dissonance occurs when a person recognizes post-decisionally that two things (e.g., one’s attitudes and choices) are not psychologically consistent. In response, the person will change her perceptions to make these attitudes and choices more consistent with each other. Feelings of discomfort the decision maker experiences post-choice arise from this inconsistency between the decision maker’s attitudes and choices, feelings the individual successfully reduces by changing his or her attitudes [21–22].

Although related in terms of conflict reduction, coherence shifting and cognitive dissonance reduction differ importantly on the timing of the preference shifts. Theories of cognitive dissonance posit that preference changes occur *after* the choice is made [18–19]. Coherence shifting emphasizes changes in assessments that begin *prior* to the choice commitment and that actually assist the person in arriving at a decision. To our knowledge, no prior research has sought to determine whether reducing emotional discomfort is indeed a driver of pre-decisional coherence shifting.

Let us return to the decision problem sketched in Table 1. If you choose the Splendor job offer, you can expect to enjoy the experience of working in a private office and having a short commute. On the other hand, taking the Splendor job would also require you to live with a lower salary and a minimal vacation package. You would have complementary good and poor experiences if you took the Bonnie’s Best job. Thus, it appears that, no matter which alternative you choose, you are condemned to suffering from your decision, comparatively speaking. There is empirical evidence that managing and potentially having to “make do” with such unresolved attribute conflict is among the major reasons why people experience some decision problems as “hard” rather than “easy” [23]. We therefore proposed that, for some people, coherence shifting allows them to avoid or assuage (not necessarily deliberately or consciously) the emotional discomfort created by attribute conflict, which also facilitates the process of arriving at a decision. The studies described here were intended to provide evidence bearing on that possibility.

### Overview of Present Studies

We predicted that attribute conflict would generate aversive emotions (Study 1), and that people who coherence shift would show reductions in the negative arousal generated by attribute conflict (Study 2). Our next goal was to collect data testing the question of whether coherence shifting is in fact an emotion regulation process. We predicted that coherence shifting would be disrupted when people were deprived of regulatory resources and would also be associated with trait level emotion regulation tendencies (Study 3). Together these studies provide, to the best of our knowledge, the first empirical evidence that attribute conflict is unpleasant, and that coherence shifting reduces these aversive emotions before a choice is made.

## Study 1: Is Attribute Conflict Aversive?

Our first goal was to establish that attribute conflict, a common feature of difficult multiattribute decisions, is indeed unpleasant. This is critical to our argument because if attribute conflict itself does not induce negative affect, then coherence shifting could not be a strategy through which individuals regulate their discomfort. To examine this issue, we manipulated attribute conflict and then assessed whether and how decision maker self-reports of negative emotions were affected by such manipulations. We hypothesized that greater attribute conflict would lead individuals to self-report more aversive negative emotions (e.g., stress, anxiety, unpleasantness), than other negative emotions (e.g., anger, sadness) or positive emotions (e.g., excitement, happiness).

## Materials and Methods

A total of 252 people from the United States, mean age = 27.3 years, 153 women, served as participants in the study. They were recruited and took part in the study via Amazon’s Mechanical Turk, for a fee of $.40. Each participant was randomly assigned to one of five conditions. The mean amount of time participants required to complete their tasks was 4 minutes. Five subjects were excluded for failing an attention check, leaving 247 cases for analysis. Procedures for this study and all subsequent studies in this paper were approved by the University of Michigan Health Sciences and Behavioral Sciences Institutional Review Board. Participants read a consent form online and proceeded with the study only if they agreed to participate.

Each participant assumed the role of a new college graduate considering job offers from department store companies “Splendor” and “Bonnie’s Best,” neither of which dominates the other. Table 1 displays the entire “Base” decision situation, from which attribute conflict was either increased or decreased. Table 3 describes the attribute conflict design as well as the decision alternatives presented to participants in each of five conflict conditions. Start with the “Base” situation in the middle of Table 3. The “Commute” and “Salary” rows in Table 3 represent the dimensions used in manipulating attribute conflict across the conditions of the experiment. In the Base situation, note that Splendor has a 22-minute advantage over Bonnie’s Best with respect to the Commute dimension. Countering that fact, Bonnie’s Best has a $1400 Salary advantage over Splendor. These aspects of the attribute conflict in the Base situation are highlighted at the top of the Base section of Table 3 to facilitate comparisons with the other conditions.

**Table 3:** Decision Matrices Varying in Attribute Conflict Intensity, Study 1.

Attribute Dimension	Condition/Attribute Conflict Intensity									
	Minus 2		Minus 1		Base		Plus 1		Plus 2	
	Splendor Adv: 4 min. Vs. Bonnie’s Best Adv: $400		Splendor Adv: 14 min. Vs. Bonnie’s Best Adv: $600		Splendor Adv: 22 min. Vs. Bonnie’s Best Adv: $1400		Splendor Adv: 30 min. Vs. Bonnie’s Best Adv: $2200		Splendor Adv: 38 min. Vs. Bonnie’s Best Adv: $3000	
	N = 51		N = 49		N = 49		N = 48		N = 50	
	**Splendor**	**Bonnie’s Best**	**Splendor**	**Bonnie’s Best**	**Splendor**	**Bonnie’s Best**	**Splendor**	**Bonnie’s Best**	**Splendor**	**Bonnie’s Best**
**Office**	Private	Cubicle	Private	Cubicle	Private	Cubicle	Private	Cubicle	Private	Cubicle
**Commute**	27 min	31 min	22 min	36 min	18 min	40 min	14 min	44 min	10 min	48 min
**Salary**	$39,900 (< $4K Std)	$40,300 (> $4K Std)	$39,800 (< $4K Std)	$40,400 (> $4K Std)	$39,400 (< $4K Std)	$40,800 (> $4K Std)	$39,000 (< $4K Std)	$41,200 (> $4K Std)	$38,600 (< $4K Std)	$41,600 (> $4K Std)
**Vacation**	Minimal	Superior	Minimal	Superior	Minimal	Superior	Minimal	Superior	Minimal	Superior

Observe that, in an ordinal fashion, the intensity of the attribute conflict implicit in the situations described in Table 3 increases from the left of the display to the right. The Commute-vs.-Salary attribute conflict in the Minus 2 condition is rather slight while that in the Plus 2 condition is substantial. In the former, a 4-minute commute time advantage is pitted against a $400 annual salary advantage. In the latter, the parallel competing advantages are much greater—38 minutes of commute time (each way, every working day) and a $3000 per year salary boost. Observe that the conditions to the left of the Base condition are labeled “Minus” situations because the intensity of attribute conflict they contain is less than that in the Base situation. The rationale for the descriptor “Plus” is analogous. Note that decisions are predicted to be less aversive when the conflict between the attributes is low because the decision maker is not giving up as much when making the choice.

In order to assess potential self-reported unpleasantness experienced by participants, we developed a decision situation *aversiveness index* based on responses to the following items presented to the participant while he or she was deliberating a particular decision problem:

**Anxious:** Rate how anxious you feel as you try to make up your mind about which of these job offers to accept: *1 = Not Anxious At All … 5 = Moderately Anxious … 9 = Extremely Anxious*

**Stressed:** Rate how stressed you feel as you try to make up your mind about which of these job offers to accept: *1 = Not Stressed At All … 5 = Moderately Stressed … 9 = Extremely Stressed*

**Unpleasant:** Rate how unpleasant it feels as you try to make up your mind about which of these job offers to accept: *1 = Not Unpleasant At All … 5 = Moderately Unpleasant … 9 = Extremely Unpleasant*

**Conflicted:** Rate how conflicted you feel as you try to make up your mind about which of these job offers to accept: *1 = Not Conflicted At All … 5 = Moderately conflicted … 9 = Extremely Conflicted*

A given participant’s aversiveness index score was that individual’s mean response, from 1 to 9, to these four items. The reliability of the aversiveness index was satisfactory: Cronbach α = .805.

### Procedure

Details of the job offers are described in Table 3. After being told these facts—first in paragraph form and then in matrix form—the participant was presented with the emotion rating task: “Before you make up your mind about which job to accept, we would like you to respond to a few statements. Keep the Splendor and Bonnie's Best job offers in mind as you respond to these statements. Please use the full range of each scale when indicating your response.” The participant responded to the four aversiveness index items as well as similarly worded items assessing negative (sadness, anger, pain, difficulty) and positive (happiness, excitement) states. We chose four specific items for the aversiveness scale because these items (i.e., anxious, stress, unpleasant, conflicted) are tied theoretically to our proposed mechanism of a negative affective state related to stress or anxiety, with the underlying appraisals being characterized by relatively higher arousal and relatively more uncertainty (cf. [24]). In this case, the appraisal of uncertainty is relevant to the future state of what the outcome might be if a person chooses one option over the other. The other negative items (sadness, anger, pain, difficulty) differ from the relatively more uncertain states related to stress or anxiety in that they are focused on a reaction to an outcome or event that has already occurred, or that a person anticipates with some certainty will occur. The positive items of happiness and excitement were chosen as comparison states because they reflect a heightened arousal state, which we predicted would also be relevant to our subset of aversive items.

## Results and Discussion

**Fig 1:**
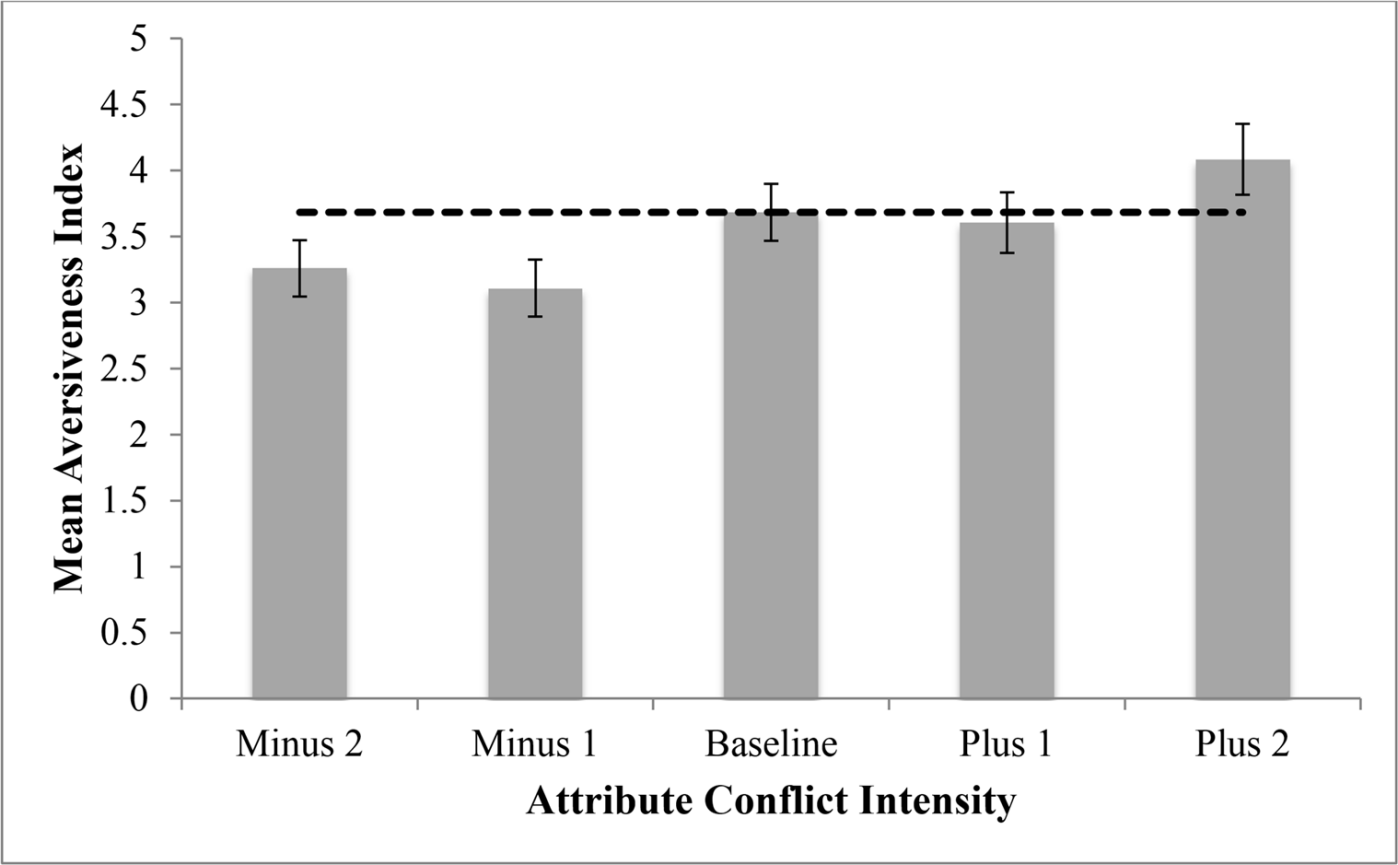
Mean aversiveness index values by attribute conflict intensity (with standard errors), Study 1. Note: 1 = “Not at All,” … 9 = “Extremely.”

Fig 1 displays the results of interest, which were consistent with our predictions. First of all, there was a statistically significant overall effect of attribute conflict intensity on aversiveness index scores, *F*(4, 242) = 2.784, *p* = .027, η^2^ = .03. The following planned contrast for mean aversiveness index scores by condition was examined: (Plus 1 + Plus 2)–(Minus 1 + Minus 2). That contrast was also statistically significant: *t*(242) = -3.05, *p* = .003, η^2^ = .04. None of the other six affective state ratings differed systematically across attribute conflict intensity. The individual results for all ten emotion items can be viewed in S2 Appendix.

Fig 1 and the effect size measures suggest that the influences of attribute conflict were small. This is likely attributable to the fact that the situations in Studies 1 and 2 were entirely hypothetical; there were no real jobs on the line to stimulate intense emotional reactions. Nevertheless, even given these constraints, we still observed significant increases in aversiveness as attribute conflict increased.

## Study 2: Associations Between Coherence Shifting and Arousal

After having established that difficult decisions involving attribute conflict are indeed aversive, we next wanted to demonstrate that coherence shifting would reduce conflict-induced negative arousal before an individual commits to a choice. If our proposal is correct, then the more an individual coherence shifts, the less emotional discomfort that person should experience over the course of making a decision. Specifically, we predicted that coherence shifting and the physiological arousal generated by emotional discomfort in multiattribute decision making would be inversely related.

## Materials and Methods

Fifty-eight undergraduates at the University of Michigan volunteered in return for course credit or a $10 fee. Participants read and signed a written consent form.

The methods were adapted from those described by Simon et al. [1] and discussed in the introduction of this article, administered via computer. The stages of the procedure, along with the materials, are summarized in Table 4. Here we provide an overview.

**Table 4:** Study 2 Procedure Sequence and Materials.

Time Point	Stage	Activity
	1	Skin conductance response (SCR) apparatus set up, palm of non-dominant hand (Biopac MP150, Biopac Systems, Goleta, CA)
	2	Baseline SCR assessment during handwriting judgment exercise
	3	Assignment to condition: Standard vs. justification expectation
	4	Generic job search scenario
1	5	Time 1 decision tasks—generic situation (E-prime Version 2.0 (Psychology Software Tools, Inc., Pittsburgh, PA): (a) -5—+5 desirability ratings (attribute appraisals), (b) 0–8 importance ratings (dimension importance weights)
	6	Distraction Task A: General knowledge questions
	7	Choice postponement scenario—potential company buyout and rescinding of Splendor or Bonnie’s Best offer
2	8	Time 2 decision tasks—Splendor vs. Bonnie’s Best situation: (a) desirability ratings (attribute appraisals), (b) importance ratings (dimension importance weights), (c) current choice leaning, (d) choice confidence, (e) preference strength
	9	Distraction Task B: Preference for how to receive health decision information
3	10	Time 3 decision tasks—Splendor vs. Bonnie’s Best situation-no buyout: (a) final choice, (b) choice confidence, (c) preference strength, (d) desirability ratings, (e) importance ratings
	11	SCR apparatus removed
	12	Final choice difficulty rating: 1—“Very Easy” *→* 7—“Very Difficult”
	13	Individual difference measures: (a) Melbourne Decision Making Scale (MDM) [25], (b) Spielberger Trait Anxiety Scale (STAI-T) [26], (c) Maximization Scale (Max) [27], (d) Obsessive-Compulsive Inventory (OCI) [28].

Note. Basic procedure and behavioral measures from Simon et al. [1].

**Stage 1.** We took as our measure of discomfort participants’ skin conductance responses (SCR), similar to the approach employed by Croyle and Cooper [21] for assessing emotional distress post-choice. Skin conductance responses were recorded via electrodes that were attached to the palm of the non-dominant hand, which remained inactive throughout the experiment. The system used was a Biopac MP150, and SCR sample acquisition was set to 500 Hz (samples/second).

**Stage 2.** To establish each participant’s SCR baseline, SCR was measured during a 5-minute handwriting judgment exercise. On each trial, the participant’s task was to provide a probability judgment for the gender of the person who wrote a short, randomly selected handwriting sample.

**Stage 3.** A priori, it seemed plausible that effects on coherence shifts might be stronger when a person anticipates having to justify his or her decision to another, respected individual (cf. [29]). Thus, participants were randomly assigned to either a “standard” condition, adapted from Simon et al. [1], or a “justification” condition that was identical except that each participant was told to imagine that he or she would have to justify the impending decision to a close other (e.g., relationship partner, parent, best friend) in a short post-choice speech. We found no effect of this manipulation on coherence shifting or choices. Although other research has found that accountability can influence pre-decisional shifting behavior (cf. [30]), the samples of interest were comprised of working professionals and subjects interacting with each other in dyads. In our study, participants were undergraduate students who were tested one-on-one and interacted only with an experimenter. Additionally, in line with the present findings, Svenson et al. [31] showed that justification instructions were associated with smaller or no consolidation effects. Future research should explore the boundary conditions of choice justification effects on coherence shifting.

**Stage 4.** The participant was asked to imagine being in a post-graduation job search situation as described in the introduction.

**Stage 5 (Decision Time 1).** This was the first point, Time 1, at which the participant reported his or her attribute appraisals and dimensional importance weights. Attribute appraisals were rated on an 11-point scale ranging from -5 (highly undesirable) to +5 (highly desirable). Dimensional importance weights were rated on a 9-point scale ranging from 0 (no weight) to 8 (maximum weight) for each of the four dimensions (i.e., salary, commute, office, and vacation). Each attribute was presented individually on the screen until the participant responded. Participants made assessments in blocks whereby they provided all of the desirability ratings first and then all of the importance weights.

**Stage 6.** This first distraction task was intended to divert the participant’s attention from the appraisals and weights just provided.

**Stage 7.** Here the participant learned of a complication in the scenario: Splendor and Bonnie’s Best were being considered for purchase by another, larger company, in which case one of the job offers might be rescinded. The experimental design objective was to delay the participant’s final decision.

**Stage 8 (Decision Time 2).** This was the first opportunity, at Time 2, for the participant to exhibit coherence shifting from the attribute appraisals and dimension weights reported at Time 1 in Stage 5. It also allowed the participant to indicate a tentative “choice leaning” between the job offers.

**Stage 9.** This healthcare decision making task was another occasion when the participant’s attention was purposely drawn away from his or her prior decision-related responses.

**Stage 10 (Decision Time 3).** In this stage, at Time 3, the participant learned that neither company would be bought out and thus each job offer remained available and a final decision and related assessments were required.

**Stage 13.** Several individual difference measures were acquired to explore theoretically plausible correlates of coherence shifting.

## Results and Discussion

### Coherence Shifting Measurement and Classification

Simon et al. [1] created separate measures of coherence shifting for each of eight attribute desirability ratings (DRs), i.e., attribute appraisals, and for each of four dimensional importance ratings (IRs), interpreted as importance weights. They observed some degree of consistency among those measures. Not surprisingly, there was considerable variability, too. To address the comprehensiveness and stability challenges, we modified and extended the Simon et al. [1] measurement approach. We first computed coherence shifting scores separately for all DRs and all IRs, which included calculating a normalized absolute measure of coherence shifting for both DRs and IRs. We then combined them to construct an overall composite coherence shifting index that we used throughout subsequently. The specific steps in creating this composite measure of coherence shifting (i.e., NACS_Overall_) are described in S1 Appendix.

### Mean Skin Conductance Responses Per Coherence Shifting

Participants’ mean SCR readings across each period of interest were interpreted as indicators of arousal and as potential proxies for emotional discomfort. Such readings were recorded and averaged for the 5-min baseline period. As expected, the correlation between coherence shifting and baseline SCR was not significant, *r*(58) = .119, *p* = .375. Thus, there was no reason to think that coherence shifting tendencies reflect chronic arousal levels.

According to the review by Figner and Murphy [32], the onset of SCRs after cognitive stimulus presentation generally occurs in the range of 1–3 seconds. Thus, we calculated a cumulative moving average from 1000 to 3000 ms after onset of the screens where participants rated desirability and importance during pre-choice Time 1, pre-choice Time 2, and post-choice Time 3.

We wanted to determine whether coherence shifting predicted changes in SCRs by assessment time—Time 1, Time 2, and Time 3—using a multilevel modeling analysis. SCR was allowed to vary by time. The linear (-1, 0, 1) and quadratic (1, 2, 1) effects of time were entered as predictors of SCRs. None of the main effects in the model were significant (linear time: *t* = -1.45, *p* = .15; quadratic time: *t* = 1.58, *p* = .12; coherence shifting: *t* = .70, p = .51). A significant coherence shifting × linear time interaction emerged predicting SCR, *b* = -.007, *SE* = .003, *t* = -2.23, *p* = .027, 95% CI = (-.014, -.0008), and the coherence shifting x quadratic time interaction was also significant, *b* = -.004, *SE* = .002, *t* = -2.50, *p* = .014, 95% CI = (-.006, -.0007). This suggests a strong relationship between coherence shifting and physiological arousal over time, with a decrease in SCRs from the pre-decisional Time 1 rating to the pre-decisional Time 2 rating, paired samples t-test: *t*(57) = 2.03, *p* = .048, 95%CI = (.0002, .0376). Importantly, the decrease in arousal from the pre-decision Time 2 rating to the post-choice Time 3 rating was not significant, paired samples t-test: *t*(57) = -.40, *p* = .70, 95%CI = (-.021, .014).

**Fig 2:**
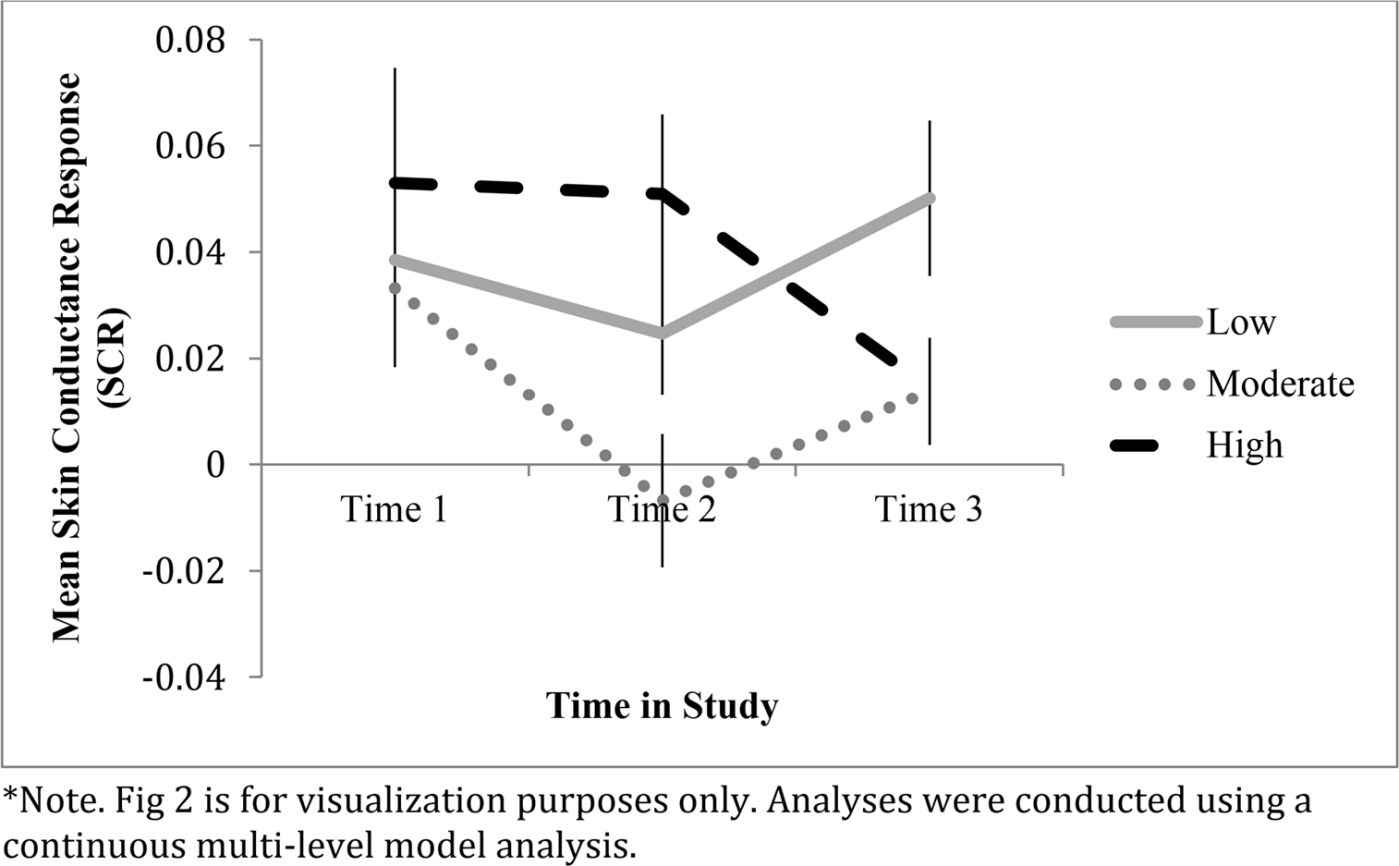
Mean skin conductance response (SCR), in μS, by coherence shifting (low, moderate, high) at 2000 ms, Study 2. Time 1 and Time 2 are “pre-choice” rating periods, while Time 3 is “post-choice.” (Bars represent standard errors of the mean.)*

To aid in understanding the relationship between coherence shifting and physiological skin conductance response (SCR), Fig 2 shows the mean values of the SCRs, distinguished by coherence shifting magnitude classification—low, moderate, and high—and by assessment time—Time 1, Time 2, and Time 3. This figure was created to more simply depict the relationship between coherence shifting and SCR, but only continuous MLM analyses were conducted on the data. As Fig 2 suggests, although there were no significant differences at Time 1 among coherence shifting groups, differences did emerge by Time 2 and Time 3, such that moderate coherence shifters reduced their arousal by Time 2, and high coherence shifters reduced their arousal by Time 3. Low coherence shifters did not reduce their physiological arousal over the decision. In other words, low coherence shifters’ arousal levels remained substantial and essentially unchanged at all three time points. In contrast, both moderate and high coherence shifters eventually achieved reductions in arousal.

**Fig 3:**
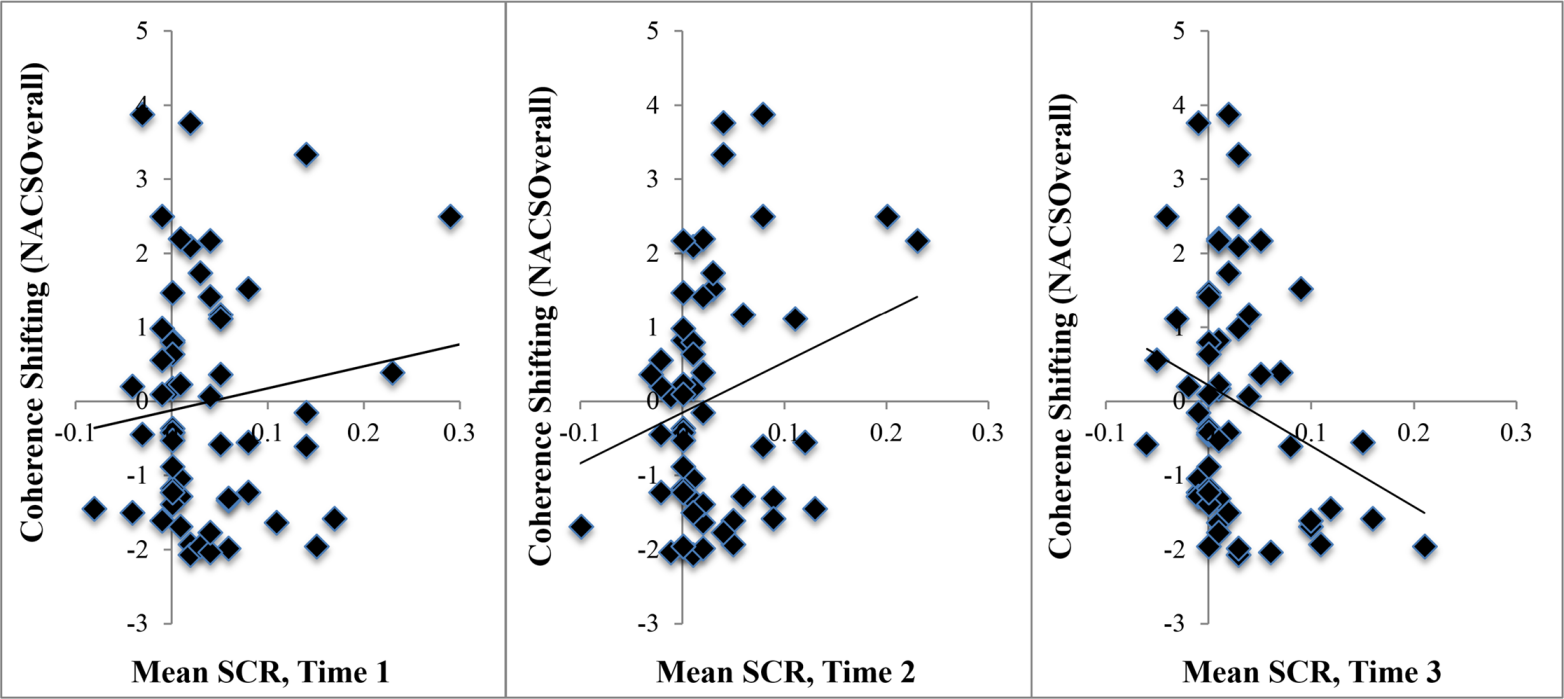
Scatterplots depicting the association between coherence shifting (NACSOverall) and mean skin conductance response (SCR), in μS, at each of three time points. Time 1 and Time 2 are “pre-choice” rating periods, while Time 3 is “post-choice.”

To examine the continuous relationship between coherence shifting and physiological arousal, Fig 3 depicts the associations between coherence shifting and SCR at each of three time points, pre-choice Time 1, pre-choice Time 2, and post-choice Time 3. As can be seen in the scatterplots, a positive association emerged from the pre-choice Time 1 (*r*(58) = .13, *p* = .318) to the pre-choice Time 2 (*r*(58) = .26, *p* = .049) rating periods, and then reversed in the post-choice Time 3 (*r*(58) = -.27, *p* = .040) rating period. This suggests an early positive association between coherence shfiting and physiological arousal that shifted to an inverse relationship by Time 3. This pattern can be better understood by referencing Fig 2, as the high coherence shifters maintain their arousal at Time 2, whereas the moderate coherence shifters experience reduced arousal by Time 2. Both moderate and high coherence shifters have acheived reduced arousal by Time 3.

### Correlates of Coherence Shifting

As predicted, the more that participants coherence shifted, the less difficult they reported the decision to be post-choice, *r*(58) = -.34, *p* = .010. This, too, supports the possibility of coherence shifting being a mechanism for managing the discomfort induced by attribute conflict.

Only one of the assessed individual difference measures was significantly correlated with coherence shifting: The less participants coherence shifted, the more they reported being “hypervigilant” on the hypervigilance subscale of the Melbourne Decision Making Questionnaire, *r*(56) = -.31, *p* = .021. Consider a brief characterization of the hypervigilance concept provided by Mann et al. [25]: “The decision maker searches frantically for a way out of dilemmas … hypervigilance is a ‘panic-like’ state in which the decision maker vacillates between unpleasant alternatives. Hypervigilance is associated with severe emotional distress.” Thus, one might reasonably speculate that people who coherence shift less perceive attribute conflict to be a distressing “dilemma” that they are unable to resolve or find a means of escaping.

## Study 3: Coherence Shifting, Emotion Regulation, and Resource Depletion

Together, Studies 1 and 2 provided evidence consistent with our proposition that coherence shifting can reliably reduce the emotional discomfort instigated by difficult decision problems involving attribute conflict. To better understand coherence shifting as a possible regulation strategy, we now turn to an examination of the processes that might disrupt coherence shifting. If diminishing people’s ability to regulate their emotions decreases coherence shifting, then this will provide convincing evidence that regulation is important to the coherence shifting process. This is especially important given that in Study 2 we found that coherence shifting was associated with lower physiological arousal by the end of the decision. If it is true that coherence shifting is an emotion regulation strategy, then it is plausible that people who coherence shift more will need to have regulatory resources available in order to do so. Consequently, if depleted of these regulatory resources, people may not be capable of applying coherence shifting as a regulatory process. Thus, the first aim of Study 3 was to begin examining whether depleting regulatory processing resources would disrupt coherence shifting.

Previous research has shown that the execution of emotion regulation activities often draws so heavily on executive control resources that meeting subsequent emotion regulation and cognitive control challenges suffers accordingly [33–36]. Resource depletion effects are in fact frequently elicited by presenting individuals with one difficult, frustrating task and observing performance decrements on a subsequent difficult, frustrating task [37]. This is because the frustrating task depletes individuals of the regulatory and motivational resources necessary to effectively engage in subsequent difficult or frustrating tasks. Similarly, we predicted that if a decision maker is already frustrated before the attribute-conflicted decision problem presents itself, then that frustration will lead to resource depletion that diminishes coherence shifting, much like other cognitive control and emotion regulation processes. After all, many of the necessary resources will have been preempted by the demands of that prior frustration. This is not to say that frustration and depletion are one and the same, but rather that the frustration evoked by difficult tasks is one means through which an individual may become depleted of regulatory resources.

In addition, you may recall from Study 2 that participants who coherence shifted reduced arousal over the course of the decision task. If these reductions in arousal are in fact indicative of emotion regulation strategy use, then we should also observe correlations between coherence shifting inclinations and general emotion regulation tendencies. There is compelling evidence that individuals sometimes consistently adopt either of two broad approaches to regulating unpleasant emotions [38–40]. A common response-focused regulation approach, referred to as “emotional suppression,” occurs when a person inhibits the expression of affect that is already being experienced. When using this strategy, it is as if the person reasons, “If I don’t show it, this bad feeling will just go away.” This regulation strategy is often observed in high-arousal emotional situations [38]. An alternative regulation approach, often described as antecedent, is called “cognitive reappraisal.” It occurs when a person reframes the situation at hand in order to preclude or quickly change negative feelings. Here, the individual might say, “If I think about this situation differently, I might realize that there’s actually no reason for alarm.” This regulation strategy tends to be selected when individuals are experiencing low-arousal emotional states [41] that are relatively easy to reappraise. The data in Studies 1 and 2 were consistent with the idea that coherence shifting can serve to prevent or reduce the emotional discomfort arising from the attribute conflict inherent in many challenging decision problems. This suggests the hypothesis that coherence shifting is a special case of the kinds of emotion regulation tools that for some time have been known to exist.

To the extent that this hypothesis is true, it is reasonable to expect that personal tendencies for exhibiting coherence shifting should be also associated with tendencies for invoking more general emotion regulation devices. Thus, in Study 3 our second aim was to provide evidence as to whether coherence-shifting tendencies are, in fact, associated with tendencies toward the use of emotion regulation tactics generally.

### Pretest

A pretest was conducted to design an anagram task that would successfully induce different levels of resource depletion. We sought to induce resource depletion through the manipulation of ambient discomfort—a kind of “incidental affect”—at varying levels [42–43]. This was achieved via anagrams rated on their difficulty and solvability as determined by prior research [44–45]. Eighteen anagrams at different challenge levels (i.e., six easy, six medium, and six difficult) were selected. The manipulation instructions were modeled after prior research using anagrams to induce discomfort [46]. Participants in the high depletion condition were instructed as follows: “Many people find this task to be easy, so you will likely not have trouble completing the task in the time allotted.” This procedure induced discomfort because participants actually struggled with the fairly difficult task that they believed others found to be easy. Participants in the low depletion condition were told: “Many people find this task to be difficult, so you will likely have trouble completing the task in the time allotted.” Participants in the baseline condition did not complete an anagram task (or any other task). Twenty-nine undergraduates (mean age = 18.5) were randomly assigned to either the low depletion or the high depletion anagram condition and rated their discomfort on a scale ranging from 1–7. Results indicated that participants in the high depletion condition (*M* = 4.78, *SD* = 1.62) self-reported more discomfort than those in the low discomfort condition (*M* = 3.53, *SD* = 1.45), *t*(27) = 2.18, *p* = .038, *d* = .8.

## Materials and Methods

One hundred and fifteen undergraduates were recruited from the University of Michigan to participate in a 30-minute experiment in exchange for course credit. Each participant was randomly assigned to a low depletion (*N* = 38), high depletion (*N* = 37), or baseline control (*N* = 40) anagram condition. The ostensibly unrelated decision was identical to that in the job offer procedure described in Study 2.

Post-decision, the participant provided demographic information and also completed the Emotion Regulation Questionnaire (ERQ) [40] and the Berkeley Expressivity Questionnaire (BEQ) [39]. Recall that in cognitive reappraisal approaches, the person tries to manage unpleasant emotions by re-construing the given situation such that any potential emotional impact is lessened [40], as articulated in an item such as, “I control my emotions by *changing the way I think* about the situation I’m in.” In contrast, expressive (emotional) suppression entails the person actively inhibiting the display of an emotion being experienced currently [40], e.g., “I control my emotions by *not expressing them*.” The negative expressivity scale of the BEQ assesses the degree to which a person tends to exhibit outward displays of experienced negative emotions, e.g., “Whenever I feel negative emotions, people can see exactly what I am feeling.” The aim of the positive expressivity scale is similar except applied to positive emotions [39], e.g., “When I'm happy, my feelings show.” Upon completion of the study, participants were debriefed about the anagram task involving deception and were awarded course credit.

## Results and Discussion

A manipulation check was given at the end of the study to determine whether each participant could recall whether the anagram task was described as easy or difficult. Participants who selected the incorrect answer were excluded for failing the manipulation check (*N* = 5, *N* = 3, and *N* = 1 in the low, high, and baseline depletion conditions, respectively). This left 106 cases for analysis. The degree of coherence shifting was calculated using the same procedures described in S1 Appendix.

### Relationship Between Resource Depletion and Coherence Shifting

**Fig 4:**
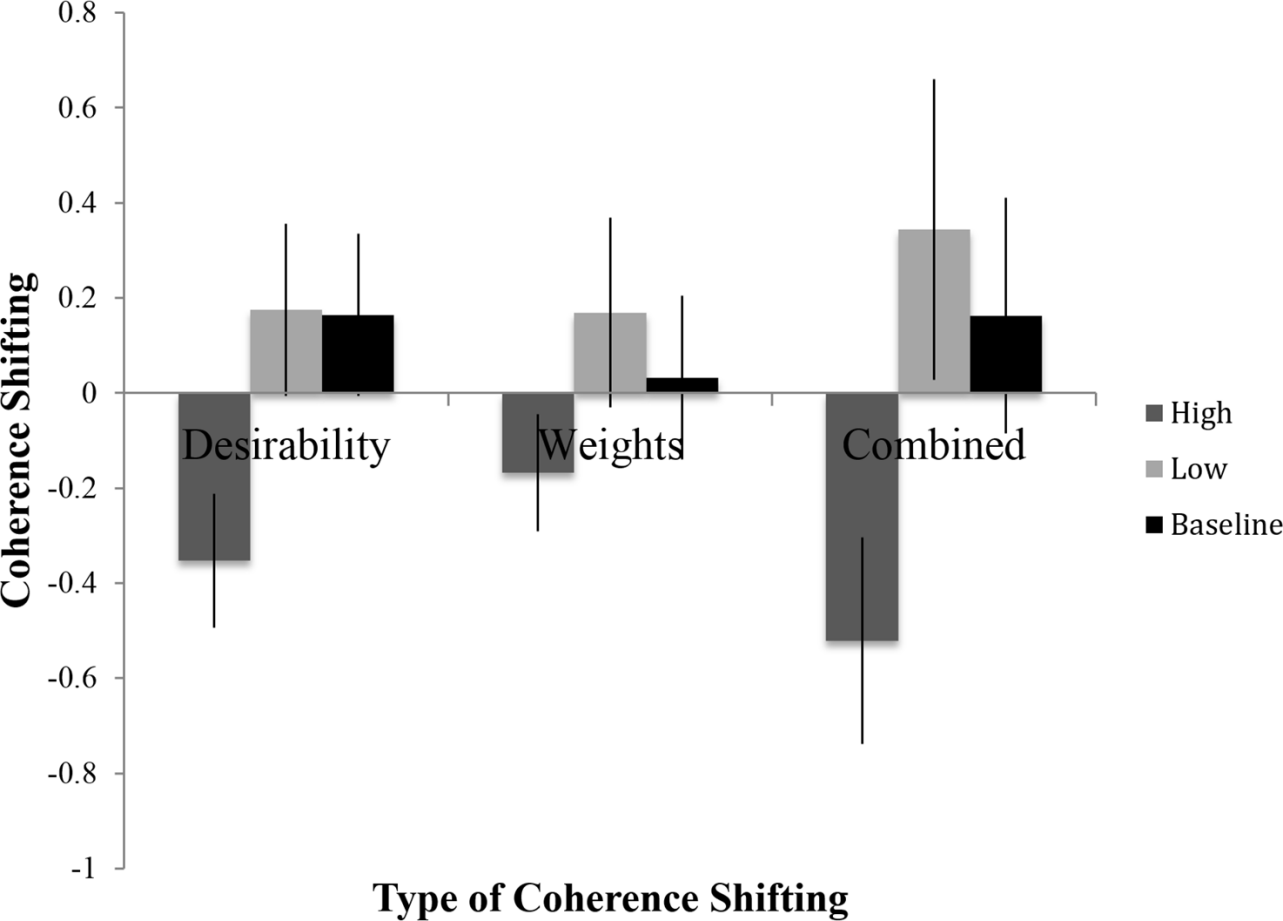
Mean coherence shifting scores for desirability ratings, dimension importance weights, and combined (i.e., NACS_Overall_), per manipulated resource depletion via ambient discomfort level—high, low, and baseline (with standard errors), Study 3.

From left to right, Fig 4 displays the mean values of the three measures of coherence shifting described in S1 Appendix: for desirability ratings (NACS_Des_), for importance weights (NACS_Imp_), and overall (NACS_Overall_). The pattern was similar for all three measures, with coherence shifting being greatest for the baseline condition and least for the high discomfort-induced depletion condition.

With respect to coherence shifting on desirability ratings, participants in the high depletion condition shifted less (*M* = -.35, *SD* = .82) than those in the low depletion (*M* = .17, *SD* = 1.03) or baseline (*M* = .16, *SD* = 1.05) conditions, *F*(2,102) = 3.26, *p* = .042, *η*^*2*^ = .060. There were no statistically reliable effects of depletion on coherence shifting of the dimension importance weights, *F*(2,103) = .954, *p* = .389. An ANOVA indicated that there was only a marginally significant influence of depletion on composite, overall coherence shifting, *F*(2,103) = 2.927, *p* = .058, *η*^*2*^ = .053. In sum, there was some evidence of an effect of depletion on coherence shifting, mainly localized to desirability ratings. What is most important, though, is the pattern. It is consistent with the assumption that coherence shifting functions like certain previously documented self- and emotion- regulation tools. In particular, the data suggest that the use of coherence shifting in a decision task is diminished when a person is faced with incidentally experienced negative emotions that presumably must first be managed or otherwise addressed. This procedure, like many other resource depletion tasks, leaves the individual devoid of the resources necessary for the decision problem, and thus disrupts the coherence shifting process.

### Associations Between Coherence Shifting and General Emotion Regulation Tendencies

To test whether the predicted associations existed between coherence shifting and general emotion regulation tendencies, correlations were computed. Results indicated that greater overall coherence shifting across conditions was significantly and positively associated with greater emotional suppression, *r*(102) = .22, *p* = .023, but not cognitive reappraisal, *r*(102) = -.061, *p* = .545. In addition, the more participants coherence shifted, the less they reported expressing their emotions in daily life: For negative emotional expressivity, *r*(98) = -.241, *p* = .017; for strength of emotional expressivity, *r*(98) = -.23, *p* = .025; and for the overall tendency to express emotions, *r*(98) = -.214, *p* = .035. However, a significant association was not found between overall coherence shifting and the tendency to express positive emotions in this study, *r*(98) = -.081, *p* = .43. This is not unreasonable given the assumption, per Study 1, that the attribute conflict manifested in the kinds of difficult decision situations under discussion generally produces feelings of unpleasantness, even stress. These results suggest that individuals may use coherence shifting to manage negative affect and that high distress can impede one’s ability to do that adaptively.

To further pursue these proposals, we repeated the main analysis testing the effect of the depletion manipulation on coherence shifting and added participants’ emotional suppression scores as a covariate in the model. This was intended to evaluate the hypothesis that individuals in the high depletion condition who self-reported a trait tendency towards emotion suppression would coherence shift more.

As hypothesized, adding emotional suppression as a covariate in the model eliminated the inverse relationship between induced depletion and coherence shifting on the composite overall coherence shifting measure, *F*(2,101) = 2.45, *p* = .092, and also on the desirability rating measure of coherence shifting, *F*(2,101) = 2.54, *p* = .084. These results further support the conclusion that a tendency toward using emotional suppression significantly contributed to observed differences in coherence shifting across depletion conditions, and this effect was particularly prominent for shifting on desirability ratings, as opposed to importance weights.

The results of Study 3 suggested that our resource depletion procedure seems to reliably reduce the strength of coherence shifting, with respect to attribute desirability although not attribute dimension importance. On the other hand, self-reported emotional suppression was positively associated with coherence shifting regardless of any resource depletion experienced by the decision maker. An important task for future studies is to evaluate the reliability of the differential impact of resource depletion and ambient discomfort on coherence shifting for attribute desirability and importance.

Our findings are also consistent with work by Simon et al. [47] showing that shifted values often return to their original ratings as quickly as twenty minutes after a decision has been made. This transience should be expected if coherence shifting is in fact a regulation tool. After all, if an important function of coherence shifting is to provide emotional relief to the decision maker, then coherence shifting should no longer be needed once the regulation has been accomplished.

## General Discussion

This article presents, to our knowledge, the first evidence that difficult decisions involving attribute conflict generate emotional discomfort *before* the decision, and that this emotional discomfort motivates regulation strategies prior to choice commitment. Specifically, we demonstrated that pre-decisional coherence shifting serves to regulate the emotional discomfort generated by difficult multiattribute decisions. Our findings suggest that emotional discomfort is created and regulated not only post-choice, as is often implicated in research on cognitive dissonance, but begins well before the choice in made. We have also presented the first evidence that pre-decisional coherence shifting, and people’s ability to use it in the adaptive service of unconflicted choice, is significantly related to their trait tendencies to regulate their emotions in particular ways. This work makes an important contribution through not only providing evidence that attribute conflict yields emotional discomfort, but also demonstrating that particular people seek relief from their conflict-induced distress via the pre-decisional procedure of coherence shifting.

Across three studies, using psychophysiology, an attribute conflict induction, and a discomfort-inducing resource depletion manipulation, we demonstrated that individuals who tend to express their emotions openly, and seldom actively regulate feelings by suppressing their overt expression, also tend to coherence shift less. In contrast, those who do self-report suppressing their emotions coherence shift more. Such coherence shifting, in turn, appears to reduce or avoid altogether the emotional discomfort generated by the attribute conflict integral to so many real-world decision situations.

Study 1 revealed that experimentally increasing attribute conflict increased emotions associated with aversive states like stress and anxiety. Study 2 provided physiological evidence consistent with our regulation proposal, as coherence shifters perceived their decisions to be less difficult and were less aroused, at least by the end of the decision process. Less coherence shifting was also associated with self-reporting a trait tendency for frantic “hypervigilance” [17, 25]. Study 3 suggested a disruptive, depleting influence of high negative incidental affect on coherence shifting, as those induced to feel distress from an unrelated source were depleted of regulatory resources and thus coherence shifted less, in terms of desirability ratings. Such shifting was associated with the extent to which individuals reported using emotional suppression as a means of managing unpleasant affect.

We used a multi-method approach across three studies to answer complementary questions about how attribute conflict influences emotions, as well as how coherence shifting helps resolve this attribute conflict by reducing the physiological arousal associated with aversive emotional states. We also showed that regulatory resources are critical for the successful use of coherence shifting strategies. In addition, we used a combination of undergraduate (Study 2, Study 3) and Amazon Mechanical Turk (Study 1) samples located in the United States. We chose to do so as we had no expectations that differences would emerge across populations. This is because decisions about jobs and housing are ubiquitous across age ranges and gender and thus should be generalizable. Future research should examine more directly whether any population differences emerge in multi-attribute decision contexts.

Despite the demonstration of connections between coherence shifting and emotion regulation across these studies, several significant challenges remain. Perhaps the most enticing is embodied in what many would probably see as a rather surprising result: As shown in Study 3, reliance on coherence shifting was positively correlated with self-reported emotional suppression tendencies, but not cognitive reappraisal. This is surprising because, on their face, the changes that occur during coherence shifting—modifications in how the decision maker appraises the desirability and importance of various attributes of choice alternatives—seem to fit the specifications of the cognitive reappraisal idea almost exactly (e.g., “construing a potentially emotion-eliciting situation in a way that alters its emotional impact”) [33]. Such modifications seem at least one step removed from how emotional suppression is commonly characterized (e.g., “inhibiting on-going emotion-expressive behavior”) [33]. In this latter view, it is as if the decision maker’s focus is on getting rid of the bad feeling that attribute conflict has generated, not on the reality of working through the actual conflict that is driving that bad feeling. Thus, the following research challenge has emerged: Why *is* coherence shifting reliably associated with emotional suppression but not cognitive reappraisal tendencies?

These speculations bring to the fore a second major challenge for future studies concerning the time course of broader coherence shifting events. For example, consistent with the results of Studies 2 and 3, people who coherence shift less may be preoccupied with more general incidental distress (cf. [34]), and may also fall prey to the hypervigilance discussed by Janis and Mann [17] and Mann et al. [25], which leads them to engage futilely in efforts to cope with their conflict-induced distress. Future research should seek to better understand the characteristics possessed by people who coherence shift a great deal, as compared to those who shift very little. It would also be important to determine whether the pre-decisional regulation processes described here differ from post-decisional cognitive dissonance reduction solely in the timing of the preference shifts, or if other factors also distinguish between these processes. One promising future method may be to use cognitive modeling or process tracing to better examine how people are making aversive multiattribute decisions. Future research should also seek to replicate and extend our Study 3 findings whereby participants depleted of regulatory resources coherence shifted less. It is plausible, for example, that specific situational factors or attribute conflict intensities require more regulatory resources than others.

Many, and probably most, important decisions in life involve attribute conflict. Jobs that pay well often also bring high stress and long commutes. Apartments close to the beach or to popular shops and restaurants are often small, expensive, and do not have parking or walk-in closets. We usually cannot, as it were, “have our cake and eat it too.” Decision problems such as these, which entail significant attribute conflict, require flexible, adaptive decision processes. Myriad psychological phenomena have been identified that appear to help people feel comfortable with such decisions, including post-hoc rationalizing, dissonance reduction, and bolstering [17]. Most of these phenomena occur after a decision is made. However, several investigators [1, 5, 6, 10] have shown that similar effects occur during the decision process itself, in the form of coherence shifting. They have also argued compellingly that plausible contributors to those effects include mechanisms that emphasize cognitive efficiency, perhaps even automaticity. The work presented here importantly contributes to this body of literature by demonstrating that pre-decisional shifting processes also serve to regulate the emotional discomfort generated by difficult decisions.

In addition to the present basic scientific challenges, it is important to address the formidable practical issues implicit in coherence shifting and the emotion factors revealed in these studies. As suggested earlier, the existence of coherence shifting is problematic for the very “logic” of multiattribute decision analysis practices [3, 48–49], which is predicated on such notions as fixed, “true” degrees of attribute importance. The emotion regulation functions of coherence shifting, implicated in the present findings, suggest that decision makers’ emotional discomfort can and almost certainly does affect the quality of their choices negatively and consequentially. Interestingly, it seems likely that in natural settings, decision makers are oblivious to the hazard. After all, coherence shifting creates the impression that the decision makers have chosen alternatives that are exceptionally strong, indeed, nearly dominating their competitors.

## Supporting Information

S1 Appendix Coherence Shifting Measurement. (DOCX)

S2 Appendix Individual Emotion Analyses. (DOCX)
