# Supplementary material for: Regulating Emotions during Difficult Multiattribute Decision Making: The Role of Pre-Decisional Coherence Shifting
Source: PLoS One. 2016 Mar 17;11(3):e0150873. doi: 10.1371/journal.pone.0150873 (PMC4795763; doi:10.1371/journal.pone.0150873)
Supplement: S1 Appendix — (DOCX) [file pone.0150873.s001.docx]

**S1 Appendix: Coherence Shifting Measurement**

The specific steps in creating our composite measure of coherence shifting were as follows.

We first defined

$$S_{Des}= (1/8)[{DR}_{Off,S} + {DR}_{Comm,S} + {DR}_{Sal,S} + {DR}_{Vac,S}-$$

${DR}_{Off,B} - {DR}_{Comm,B} - {DR}_{Sal,B} - {DR}_{Vac,B}],$ (2)

where each rating was linearly transformed to a -1 🡪 +1 scale, per Simon et al. (2004). In this equation, DR_Off,S_ denotes the desirability rating the participant gave to the office (Off) attribute for the Splendor (S) alternative and DR_Sal,B_ represents the desirability rating for the salary (Sal) in the Bonnie’s Best (B) job offer. The remaining expressions on the right-hand side of Eq. 2 have similar meanings for the rest of the attributes. S_Des_ is the same as the statistic described as the “S score” by Simon et al. (2004). It reflects the overall attractiveness of Splendor’s attributes compared to those of Bonnie’s Best. S_Des_ was calculated for the participant’s Time 1 as well as Time 2 assessments of attribute desirability, yielding S_Des,1_ and S_Des,2_, respectively. From these, we computed

${CS}_{Des} = S_{Des,2}-S_{Des,1},$ (3)

a “raw” desirability coherence shifting measure for the participant. This allowed us to define

${ACS}_{Des} = | S_{Des,2}-S_{Des,1} |$ (4)

as an absolute measure of desirability coherence shifting. Taking the absolute value allows us to capture appropriately the magnitudes of the shifts, irrespective of their direction. Finally, we specified

${NACS}_{Des}= z({ACS}_{Des}),$ (5)

as a “normalized” measure of the participant’s desirability coherence shifting, relative to that exhibited by his or her co-participants.

In analogy with S_Des_, we defined

$S_{Imp}=(1/4)[{IR}_{Off} + {IR}_{Comm}- {IR}_{Sal} - {IR}_{Vac}],$ (6)

where IR_Off_ represents the importance rating for the office attribute dimension and the remaining expressions have similar interpretations for the other dimensions, with each rating having been transformed linearly to a 0 🡪 1 scale. S_Imp_ indexes the overall importance the participant attached to the dimensions on which Splendor was better than Bonnie’s Best, relative to those on which Bonnie’s Best was better than Splendor. Taking the same approach as with desirability, we specified

${CS}_{Imp} = S_{Imp,2}- S_{Imp,1},$ (7)

${ACS}_{Imp} = | S_{Imp,2}- S_{Imp,1} |,$ (8)

and

${NACS}_{Imp} = z({ACS}_{Imp}),$ (9)

that is, a normalized absolute measure of coherence shifting with respect to dimension importance.

Finally, as a composite measure of each participant’s extent of coherence shifting, with respect to both desirability and importance ratings, we defined

${NACS}_{Overall}= {NACS}_{Des} + {NACS}_{Imp}$ (10)
