# Supplementary material for: Regulating Emotions during Difficult Multiattribute Decision Making: The Role of Pre-Decisional Coherence Shifting
Source: PLoS One. 2016 Mar 17;11(3):e0150873. doi: 10.1371/journal.pone.0150873 (PMC4795763; doi:10.1371/journal.pone.0150873)
Supplement: S2 Appendix — (DOCX) [file pone.0150873.s002.docx]

**S2 Appendix: Individual Emotion Analyses**

These results reflect planned contrasts in Study 1 comparing the emotions generated by the Minus 1 (low attribute conflict) and Minus 2 (lowest attribute conflict) conditions versus the Plus 1 (high attribute conflict) and Plus 2 (highest attribute conflict) conditions. In other words, these analyses compared the self-reported emotion ratings for participants in the low attribute conflict conditions versus those in the high attribute conflict conditions.

Stressed: *t*(242) = 2.79, *p* = .006*

Anxious: *t*(242) = 2.17, *p* = .031*

Unpleasant: *t*(242) = 2.65, *p* = .009*

Conflicted: *t*(242) = 1.56, *p* = .121*

Angry: *t*(242) = .17, *p* = .861

Difficult: *t*(242) = .72, *p* = .470

Painful: t(242) = -.78, *p* = .435

Sad: *t*(242) = .45, *p* = .886

Excited: *t*(242) = .39, *p* = .694

Happy: *t*(242) = .14, *p* = .888

*Asterisk denotes items included in the aversiveness index.
